# Deep learning-based auto-delineation of gross tumour volumes and involved nodes in PET/CT images of head and neck cancer patients

**DOI:** 10.1007/s00259-020-05125-x

**Published:** 2021-02-09

**Authors:** Yngve Mardal Moe, Aurora Rosvoll Groendahl, Oliver Tomic, Einar Dale, Eirik Malinen, Cecilia Marie Futsaether

**Affiliations:** 1 Faculty of Science and Technology, Norwegian University of Life Sciences, Ås, Norway; 2 Department of Oncology, Oslo University Hospital, Oslo, Norway; 3 Department of Medical Physics, Oslo University Hospital, Oslo, Norway; 4 Department of Physics, University of Oslo, Oslo, Norway

**Keywords:** Deep learning, Delineation, Head and neck cancer, Automatic delineation

## Abstract

**Purpose:**

Identification and delineation of the gross tumour and malignant nodal volume (GTV) in medical images are vital in radiotherapy. We assessed the applicability of convolutional neural networks (CNNs) for fully automatic delineation of the GTV from FDG-PET/CT images of patients with head and neck cancer (HNC). CNN models were compared to manual GTV delineations made by experienced specialists. New structure-based performance metrics were introduced to enable in-depth assessment of auto-delineation of multiple malignant structures in individual patients.

**Methods:**

U-Net CNN models were trained and evaluated on images and manual GTV delineations from 197 HNC patients. The dataset was split into training, validation and test cohorts (*n*= 142, *n* = 15 and *n* = 40, respectively). The Dice score, surface distance metrics and the new structure-based metrics were used for model evaluation. Additionally, auto-delineations were manually assessed by an oncologist for 15 randomly selected patients in the test cohort.

**Results:**

The mean Dice scores of the auto-delineations were 55*%*, 69*%* and 71*%* for the CT-based, PET-based and PET/CT-based CNN models, respectively. The PET signal was essential for delineating all structures. Models based on PET/CT images identified 86*%* of the true GTV structures, whereas models built solely on CT images identified only 55*%* of the true structures. The oncologist reported very high-quality auto-delineations for 14 out of the 15 randomly selected patients.

**Conclusions:**

CNNs provided high-quality auto-delineations for HNC using multimodality PET/CT. The introduced structure-wise evaluation metrics provided valuable information on CNN model strengths and weaknesses for multi-structure auto-delineation.

**Supplementary Information:**

The online version contains supplementary material available at (10.1007/s00259-020-05125-x10.1007/s00259-020-05125-x).

## Introduction

Radiotherapy (RT) with concurrent chemotherapy is the preferred curative treatment option for inoperable head and neck cancer (HNC) [1]. An essential part of RT is tumour delineation, where the tumour and involved lymph nodes are carefully outlined in medical images. This task is vital to ensure that all malignant tissues are included in the RT treatment volume.

Positron emission tomography/X-ray computed tomography (PET/CT) is a highly useful modality for imaging and subsequent delineation of HNC for RT [2]. In most cases, CT is performed with an iodinated contrast agent [3]. Tumours and involved nodes may be detected on PET images, as these regions normally have higher metabolic activity than surrounding healthy tissue. However, PET is limited by low spatial resolution, and combining PET images with high-resolution CT images may improve delineation quality. Several studies have found a significant reduction in interobserver variability for manual gross tumour volume (GTV) delineations in HNC when using combined PET/CT instead of CT [4–7]. Despite this, considerable interobserver variations still occur. In a recent HNC interobserver study, the average overlap between PET/CT-based GTV delineations made by expert radiation oncologists was 69*%* (as measured by the Dice score) [7]. Moreover, the manual delineation process is time consuming and can be a bottleneck in RT planning. Finding methods to improve delineation quality and reduce the workload is therefore highly warranted.

Automatic tumour delineation using deep convolutional neural networks (CNNs) can potentially provide delineation consistency and time-efficiency. Recent studies show a high degree of overlap between expert’s tumour delineations and those proposed by CNNs [8–10]. There has, to date, been few studies on CNN auto-delineation of HNC lesions using multimodality images. In [8], Lin et al. used a 3D CNN to successfully auto-delineate the GTV of nasopharyngeal cancers from PET/MRI images. These delineations were evaluated both quantitatively and qualitatively, the latter by expert oncologists. Likewise, Huang et al. [9] used a 2D CNN to delineate the GTV of HNC lesions in PET/CT images. Although very promising, these studies did not consider involved lymph nodes, which, according to current practice, should be prescribed the same RT dose as the GTV. A typical patient with HNC may have multiple involved neck nodes and delineating these is essential for adequate RT [2]. This issue was addressed by Guo et al. [10] who used 3D CNNs to delineate both the GTV and involved nodes in CT, PET and combined PET/CT images. In the latter study, the quality of the network delineations was evaluated quantitatively on a patient-wise basis, regardless of the number of structures delineated.

The scoring of involved lymph nodes does, however, constitute a challenge in evaluating the quality of auto-delineations. This is apparent for occult or small lesions that have been judged as malignant by the expert but not by the auto-delineation method (false negatives). For these situations, the overall performance of the automatic method may be interpreted as poor when assessed using, for example, distance-based metrics, despite a high agreement for the tumour and larger nodes. The same problem also arises for false positive predictions, where the auto-delineation program may incorrectly delineate an hypermetabolic region as part of the GTV. In this case, the distance between this falsely delineated structure and the true GTV may be very large, even though there is high agreement between all other predicted structures and the ground truth. Thus, there is a need for standardised methods to estimate the performance of multi-structure auto-delineation, when the expert delineations include both primary tumour and involved nodes.

The aim of the current study was threefold. First, we evaluated 2D CNN models for fully automatic delineation of both the gross (primary) tumour volume (GTV-T) and the malignant nodal volume (GTV-N) in patients with HNC. Secondly, as all patients underwent a combined PET/CT examination prior to treatment, network performance was assessed using single-modality (CT or PET) as well as multimodality (PET/CT) image input, to determine which modality or modality combination provided the most accurate auto-delineations. Thirdly, we introduce a new framework for structure-wise performance evaluation of multi-structure auto-delineations, as a supplement to already well-established performance metrics. This framework provides additional metrics to quantify the similarity between the expert’s ground truth and the network predictions when more than one contoured structure is present in the ground truth, thereby enabling thorough evaluation of the strengths and weaknesses of auto-delineation approaches. Finally, auto-delineations were qualitatively assessed by an expert oncologist.

## Material and methods

### Imaging and contouring

HNC patients referred to curative chemoradiotherapy at Oslo University Hospital from January 2007 to December 2013 were retrospectively included, as described in [11]. Briefly, inclusion criteria were squamous cell carcinoma of the oral cavity, oropharynx, hypopharynx and larynx treated with curatively intended radio(chemo)therapy and available radiotherapy plans based on FDG PET/CT. Nasopharyngeal cancers were excluded, as were patients with known distal metastases and post-operative radiotherapy without residual tumour. In addition, patients without a contrast-enhanced planning CT were excluded, resulting in 197 patients included in the current analysis. Patient characteristics are provided in Supplementary Table A1 (Online Resource 1). The study was approved by the Regional Ethics Committee (REK) and the Institutional Review Board. Exemption from study-specific informed consent was granted by REK.

All patients had an RT optimised PET/CT scan (CT with contrast enhancement) taken on a Siemens Biograph 16 scanner (Siemens Healthineers Gmbh, Erlangen, Germany). After ≥ 6 h of fasting 370 ± 20 MBq FDG was injected, and the patient rested for about one hour until imaging. Image acquisition was performed on an RT-compatible flat table with head support in an RT fixation mask. PET acquisition time was 5 min/bed position with 25*%* overlap between positions. The PET coincidence data were reconstructed using the OSEM4,8 algorithm with a Gaussian post-reconstruction filter with full width at half maximum equal to 3.5 mm for 193 patients, 2 mm for 3 patients and 5 mm for 1 patient. PET pixel size varied between 1.33 and 4.06 mm (mode 2.66 mm for 143 patients) in a 256 × 256 matrix with a slice thickness of 1.0–5.00 mm (mode 2.00 mm for 169 patients). CT images were obtained with a peak tube voltage of 120 kV, giving a reconstructed matrix of 512 × 512, a pixel size of around 1.0 mm and a slice thickness of 2.0 mm. The Visipaque contrast agent was used, and the CT acquisition was performed after a delay of about 30 s post-injection. All PET and CT image series were resampled to a common isotropic 1 × 1 × 1 mm^3^ reference frame. The resulting image slices were cropped to a 191-by-265 mm^2^ axial region of interest, keeping the patient in the centre of the full image stack.

The primary tumour (GTV-T) and, if present, malignant lymph nodes (GTV-N) were manually delineated by an experienced nuclear medicine specialist, based on the FDG uptake. These delineations were further refined by one to two (of many) oncology residents based on the contrast-enhanced CT and clinical information such as the endoscopy report. The delineations were finally approved by one of several senior oncologists. All delineations were performed at the time of initial RT (i.e. the patients received RT based on these delineations). The union of the manual GTV-T and GTV-N delineations were defined as the ground truth and used for training and evaluation of the CNN models. An overview of the number of manually delineated structures per patient and their volumes is given in Supplementary Tables A2 and A3 (Online Resource 1).

### Model architecture and training

A U-Net architecture following the setup described in [12] was trained to delineate GTV-T and GTV-N in the PET/CT image slices. There was one addition to the original U-Net architecture, namely that batch normalisation [13] was applied after each ReLU non-linearity. Model details are provided in Supplementary Table A4 (Online Resource 1).

Four different loss functions were compared as follows: (1) the cross-entropy loss, (2) the Dice loss [14] and (3) the *f*_β_ loss with β ∈{2,4} [15]. For each loss function, the models were trained using CT images only, PET images only and both PET and CT images. Additionally, the impact of CT windowing on model performance was assessed, using a narrow soft-tissue window of width 200 HU and a centre of 70 HU (range: [− 30, 170] HU). The window centre of 70 HU corresponded to the median HU value within the GTV-T and GTV-N in the training set. In total, 20 models were run (i.e. 4 loss functions × (3 image input combinations without windowing + 2 input combinations with windowing)).

To assess model performance, we split the patients into three cohorts, stratifying by the primary tumour (T) stage of the TNM staging system to ensure similar patient characteristics across cohorts: A training cohort (142 patients), a validation cohort (15 patients) and a test cohort (40 patients). Patient characteristics of these cohorts are given in Supplementary Table A1 (Online Resource 1). To compare models, the patient-wise Dice score (1) was evaluated on patients in the validation cohort. Then, for each modality, the model achieving the highest Dice score was used to delineate in images from the test cohort. These test cohort auto-delineations were evaluated in depth, using the qualitative and quantitative methods described below.

To train the model, we used the Adam optimiser [16] with the β-values^1^ recommended in [16] and a learning rate of 10^− 4^. The model was trained for 20 epochs, and the network coefficients were saved to disc (checkpointed) every second epoch. After training a model, we compared the average Dice score per image slice of each coefficient checkpoint. The coefficient checkpoint with the highest slice-wise Dice was used for subsequent performance analysis.

No post-processing was applied on the model output, such that the raw delineations provided by the CNN models were assessed without modifications.

### Quantitative performance evaluation

#### Patient-wise metrics

Similarity and surface-distance metrics were used to assess the quality of the predicted delineations generated by the CNN models. Firstly, we measured overall delineation accuracy by the patient-wise (i.e. per patient) Dice score. The Dice score is given by:

1 $$ \text{Dice}(X, \hat{X}) = \frac{\left| X \cap \hat{X} \right|}{\frac{1}{2}\left| X \right| + \frac{1}{2}\left| \hat{X} \right|},  $$

where |*X*| and $|\hat {X}|$ are the number of voxels in the ground truth, and the predicted delineations, respectively, and $| X \cap \hat {X} |$ denotes the number of voxels in the intersection between the ground truth and predicted delineations.

Next, we computed three surface-distance-based metrics for each patient (i.e. patient-wise): (1) the 95th percentile Hausdorff distance (HD_95_), (2) the average surface distance (ASD) and (3) the median surface distance (MSD), all three of which were calculated from the same set of boundary distances. For a boundary voxel *i* in the predicted delineation, we computed its smallest distance *D*_*i*_ to the ground truth boundary $\tilde {X}$, given by:

2 $$ D_{i} = \min\limits_{\tilde{\boldsymbol{x}}_{j} \in \tilde{X}} \text{dist}(\hat{\tilde{\boldsymbol{x}}}_{i}, \tilde{\boldsymbol{x}}_{j}),  $$

where $\text {dist}(\hat {\tilde {\boldsymbol {x}}}_{i}, \tilde {\boldsymbol {x}}_{j})$ is the (Euclidean) distance between the predicted boundary voxel *i* with coordinates $\hat {\tilde {\boldsymbol {x}}}_{i}$ and the true boundary voxel *j* with coordinates $\tilde {\boldsymbol {x}}_{j}$. From the set of all such distances, we computed HD_95_ as its 95% quantile, ASD as its average and MSD as its median. Thus, the HD_95_ measures how severe the largest delineation error is, and the ASD and MSD measure the overall delineation error. These surface-distance metrics should be as small as possible.

#### Structure-wise metrics

**Fig. 1:**
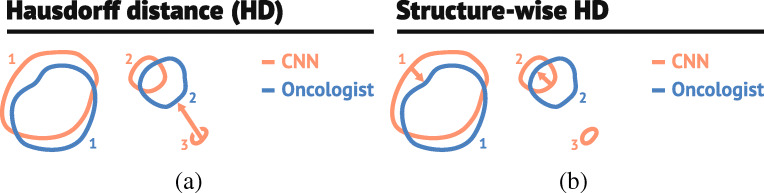
The illustration in **a** demonstrates how the surface-distance based metrics (HD $= \max \limits _{i} D_{i}$) can be non-informative in the presence of falsely predicted structures. As CNN structure 3 (*red*, CNN) does not overlap with any of the true manually delineated structures (*blue*, Oncologist), it is defined as a falsely predicted structure. Computing distance metrics between the false structure 3 and true structures will increase the metrics. The illustration in **b** shows how this problem can be alleviated by only computing the surface-distance-based metrics for predicted structures (*red*, labelled 1 and 2) that have sufficient overlap with the manually delineated ground truth (*blue*, labelled 1 and 2)

The distance-based metrics can be skewed if the CNN model misses a true structure or falsely predicts an additional structure not included in the ground truth, as illustrated in Fig. 1a. If distance-based metrics are to capture the delineation quality of the structures that are actually detected, they should be computed for true and predicted structures that overlap. Thus, we computed the degree of overlap between true and predicted structures, giving the *coverage fraction* (CFrac):

3 $$ \text{CFrac}(\hat{X}_{k}, X) = \frac{\left| \hat{X}_{k} \cap X\right|}{\left|\hat{X}_{k}\right|}.  $$

**Fig. 2:**
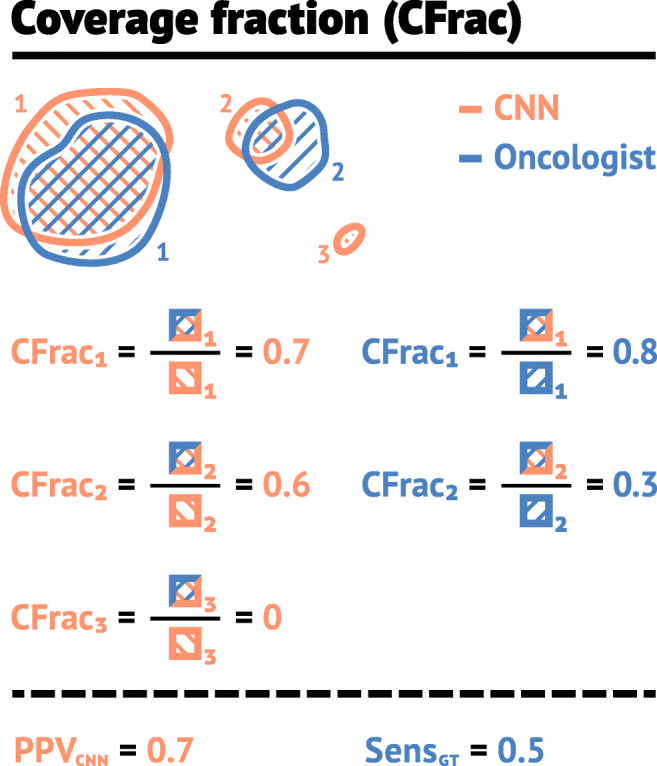
Illustration of the coverage fraction metric CFrac. The left column gives the CFrac (3) for the overlap between the predicted (*red*, CNN) and true (*blue*, Oncologist) structures relative to the CNN structure (*red*). In this case, the PPV_CNN_ is 0.7, as two of the structures (*red*, 1 and 2) proposed by the CNN-model were delineated by the oncologist, and one (*red*, structure 3) was not. The right column gives CFrac for the overlap between the CNN (*red*) and true (*blue*) structures relative to the true structure (*blue*). The Sens_GT_ is equal to 0.5 since one true structure (*blue*, 1) was identified by the CNN-model and one was not (*blue*, 2), as its coverage fraction was less than 0.5

Here $\hat {X}_{k}$ is the set of voxels in the *k* th structure of the predicted mask. An illustration of the CFrac is given in Fig. 2. If the CFrac was greater than 0.5, the predicted structure was defined as correctly identified by the CNN model. Thereafter, HD_95_, MSD and ASD were computed separately for all structures in the auto-delineation with CFrac ≥ 0.5, as shown in Fig. 1b, giving structure-wise distance metrics not skewed by falsely predicted structures.

To further assess the performance of the CNN model, we defined a structure-wise sensitivity and positive predictive value. The number of true negative structures cannot be defined, and the number of true positive structures varies according to perspective (ground truth vs auto-delineation). As illustrated by the example in Fig. 2, there are two structures (1 and 2, red) in the auto-delineated mask that obtain a CFrac above 0.5 with the ground truth, and one that does not (red structure 3). The auto-delineation, therefore, has two true positive structures and one false positive structure. Thus, we define the structure-wise positive predictive value with respect to the CNN model (PPV_CNN_) as:

4 $$ \text{PPV}_{\text{CNN}} = \frac{\text{TP}_{\text{CNN}}}{\text{TP}_{\text{CNN}} + \text{FP}},  $$

where TP_CNN_ is the number of true positive structures in the auto-delineation mask and FP is the number of false positive structures. For Fig. 2, TP_CNN_ = 2 and FP= 1, giving a PPV_CNN_= 0.7 (rounded to one significant digit).

Likewise, for Fig. 2, there is one structure (1, blue) in the ground truth that obtain a CFrac above 0.5 with the auto-delineation mask, meaning that there is only one true positive with respect to the ground truth. Consequently, we define structure-wise sensitivity with respect to the ground truth (Sens_GT_):

5 $$ \text{Sens}_{\text{GT}} = \frac{\text{TP}_{\text{GT}}}{\text{TP}_{\text{GT}} + \text{FN}},  $$

where TP_GT_ is the number of true positive structures with respect to the ground truth and FN is the number of structures in the ground truth not delineated by the CNN model (false negatives). In Fig. 2, TP_GT_ = 1 and FN= 1, giving a Sens_GT_ = 0.5.

Finally, to further assess errors made by the CNN model, we calculated (1) the volume of structures in the auto-delineation that obtained a CFrac > 0.5 with the ground truth (Volume_true_), and (2) the volume of structures in the auto-delineation that obtained a CFrac ≤ 0.5 with the ground truth (Volume_false_). For the delineations in Fig. 2, the Volume_true_ is the mean volume of CNN (red) structures 1 and 2 and Volume_false_ is the volume of CNN structure 3.

### Qualitative evaluation

The CNN model with superior mean Dice performance was qualitatively evaluated by an expert oncologist with more than 7-year experience in HNC target volume delineation. The expert was presented with the ground truth and the delineations made by the CNN-model for 15 patients randomly selected from the test cohort. The expert did not know which contour was CNN-generated and which was human-generated. For each of these patients, the oncologist was asked to identify (if possible) which delineation was generated by the CNN model. The oncologist scored the quality of the selected auto-delineation masks using a score from one to ten. A score of one represented a delineation with little to no clinical value and a score of ten represented a delineation where the oncologist was unable to identify whether the mask was generated by the CNN model or human specialists, implying high clinical value.

### Code

Models were trained using Python and TensorFlow. Code for running the experiments is provided at https://github.com/yngvem/EJNMMI-20. Performance metrics were computed using an in-house developed Python library provided at: https://github.com/yngvem/mask_stats.

## Results

### Comparison of models

The average model performance on the validation cohort is summarised in Table 1. All models had an average Dice between 0.40 and 0.65. Note that standard deviations of the Dice score and structure-wise performance metrics were relatively small, indicating that the model performance was stable between models trained with the same modality and windowing option, but with different loss functions. Thus, loss function choice had little effect on performance.

**Table 1:** The performance (mean ± one standard deviation) of CNN models trained using different modalities

			Modality				
			PET	CT		PET/CT	
			–	CTW	CT	CTW	CT
Patient-wise	Dice	(%)	61 ± 2	55 ± 2	48 ± 5	63 ± 1	62 ± 1
	ASD	(mm)	8.1 ± 2.6	11 ± 3	13 ± 7	7.0 ± 0.8	8.0 ± 3.1
	MSD	(mm)	4.5 ± 1.9	5.6 ± 0.8	7.8 ± 2.5	4.6 ± 1.0	4.6 ± 2.6
	HD_95_	(mm)	31 ± 14	38 ± 17	50 ± 44	24 ± 2	32 ± 17
	Sens_GT_	(%)	75 ± 5	60 ± 9	53 ± 11	75 ± 4	78 ± 7
	PPV_CNN_	(%)	25 ± 5	22 ± 6	21 ± 11	26 ± 4	28 ± 11
Structure-wise	ASD	(mm)	1.6 ± 0.2	2.1 ± 0.4	2.4 ± 0.6	1.6 ± 0.3	1.4 ± 0.2
	MSD	(mm)	1.1 ± 0.2	1.7 ± 0.4	1.9 ± 0.6	1.1 ± 0.3	0.92 ± 0.14
	HD_95_	(mm)	4.9 ± 0.7	5.6 ± 1.0	6.0 ± 1.0	4.5 ± 0.5	4.4 ± 0.6
	Volume_true_	(cm^3^)	17 ± 5	9.7 ± 3.7	11 ± 3	15 ± 4	16 ± 3
	Volume_false_	(cm^3^)	1.1 ± 1.3	0.41 ± 0.17	1.6 ± 2.6	0.58 ± 0.54	0.58 ± 0.41

Choice of loss function had little effect on performance, and averaging therefore was done over models trained with different loss functions. CTW and CT columns represent models trained with and without CT windowing, respectively. All models were evaluated on the validation cohort

Imaging modality and Hounsfield windowing of CT images, however, had a clear effect on performance. Models trained on both PET and CT images had the highest patient-wise Dice performance and the lowest surface distances, indicating a high degree of overlap between the model prediction and the ground truth. Models trained solely on PET images had lower Dice and larger surface distances than PET/CT models, but outperformed models based on CT images on all performance metrics. Note that patient-wise surface distances were both larger and more varied than structure-wise due to measurements between false positive structures and the ground truth.

From Table 1, it is also apparent that models, on average, identified more than 50*%* of the manually delineated structures in the validation cohort (Sens_GT_). Particularly models built using PET images had high detection rates, identifying more than 75*%* of true structures. However, the models generated many false positive structures, which is apparent from the low PPV_CNN_. On average, less than a third of all delineated structures in the CNN masks (for all modalities) were also present in the ground truth. Despite this, the Dice was high, indicating that the false positive structures were small in volume (see Volume_false_ in Table 1).

### Performance on the test cohort

**Fig. 3:**
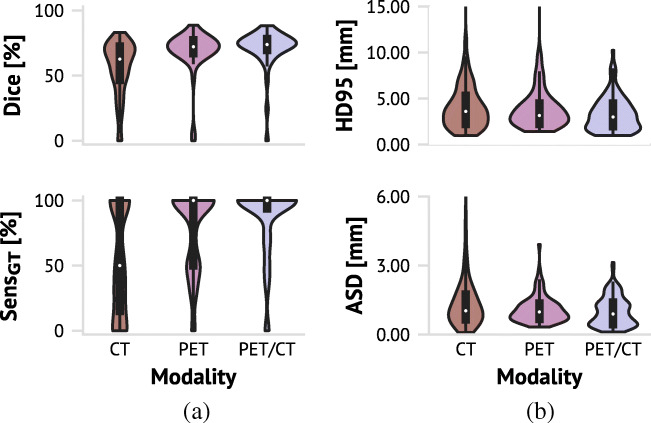
Violin plots with boxplots overlayed (*dark gray box* within) for test cohort performance metrics of models achieving the highest Dice based on each imaging modality. **a** Patient-wise Dice score and structure-wise sensitivity (Sens_GT_). **b** Structure-wise HD_95_ and ASD distance metrics. Note that the axis for ASD is cut off at 6 mm to improve visualisation for the PET and PET/CT-based models. There was one structure generated by the CT-based model which had an ASD outside this range (13 mm). Refer to Table 2 for details

For each input-modality, the model that achieved the highest average Dice score on the validation cohort was selected for further evaluation on the test cohort. The best PET-based model was trained with the Dice loss function. The best CT-based and PET/CT-based models were trained using CT-windowing and the *f*_2_ loss function. Test cohort performance metrics are shown in Table 2 and Fig. 3. The CT model had the lowest patient-wise Dice score (56*%*) as well as the largest patient-wise distance metrics, indicating poorer overlap between the predicted delineation and the ground truth relative to PET and PET/CT models. PET and PET/CT models achieved high Dice performance (69*%* and 71*%*, respectively) and structure-wise sensitivity (Sens_GT_) (77*%* and 86*%*, respectively), indicating that these models had high overlap with the ground truth and detected the majority of the manually delineated structures (i.e. few false negative structures, Eq. 5).

**Table 2:** Performance on the test cohort for the CNN models with the highest Dice score on the validation set, using different input modalities

			Modality					
			PET		CT		PET/CT	
			Mean	Std.	Mean	Std.	Mean	Std.
Patient-wise	Dice	(*%*)	69	17	56	21	71	16
	ASD	(mm)	4.2	4.3	6.1	5.1	4.7	4.8
	MSD	(mm)	1.9	4.0	3.1	5.4	1.8	4.0
	HD_95_	(mm)	18	16.5	22.2	11.7	21.2	17.1
	Sens_GT_	(*%*)	77	31	53	41	86	27
	PPV_CNN_	(*%*)	45	29	28	17	33	23
Structure-wise	ASD	(mm)	1.1	0.6	1.4	1.4	1.0	0.6
	MSD	(mm)	0.61	0.54	0.96	1.45	0.56	0.67
	HD_95_	(mm)	4.0	2.6	4.1	2.5	3.3	1.8
	Volume_true_	(cm^3^)	17	22	7.2	12	15	24
	Volume_false_	(cm^3^)	0.45	1.1	0.56	1.7	0.54	3.0

The CT images were pre-processed using windowing

Even though the CT model on average identified 53*%* of the manually delineated structures (Sens_GT_), it was unable to identify even a single structure for 10 patients in the test cohort (data not shown). In contrast, the PET and PET/CT models failed to identify a single structure for only two patients. Moreover, from the boxplots overlayed on the violin plots in Fig. 3a, we see that the 25th percentile Sens_GT_ was 15*%* for the CT-based model, while it was 50*%* for the PET-based model and 93*%* for the PET/CT-based model, again highlighting the PET/CT model’s high rate of structure identification.

The structure-wise metrics illustrate (see Table 2 and Fig. 3) that using both the PET and CT signal simultaneously was beneficial compared to only using one modality. All structure-wise surface distance metrics were smaller for the PET/CT model and spanned a narrower range with fewer large outliers compared to the models that used only a single modality. Thus, PET/CT-based auto-delineations were more accurate with fewer large deviations between the predicted and true structure boundaries.

Note also that the structure-wise distance metrics (ASD, MSD, HD_95_) were considerably smaller than the corresponding patient-wise distance metrics (Table 2). This indicates that the patient-wise distance metrics were influenced by measurements between falsely delineated structures (false positives) and the ground truth (see Fig. 1a). This is further supported by the metric PPV_CNN_, which was below 50*%*, indicating that the CNN models tended to delineate several false positive structures (i.e. large FP, Eq. 4). However, the volumes of the erroneously predicted structures (Volume_false_) were small compared to the true structure volumes (see Supplementary Table A2, Online Resource 1). This is also reflected by the high Dice score of all the models. Furthermore, the average volume of the erroneous structures (Volume_false_) for the PET/CT model was 0.54 cm^3^. There were only five true structures in the entire data set (< 5*%*) smaller than or equal to this size (data not shown).

### Qualitative performance evaluation

**Fig. 4:**
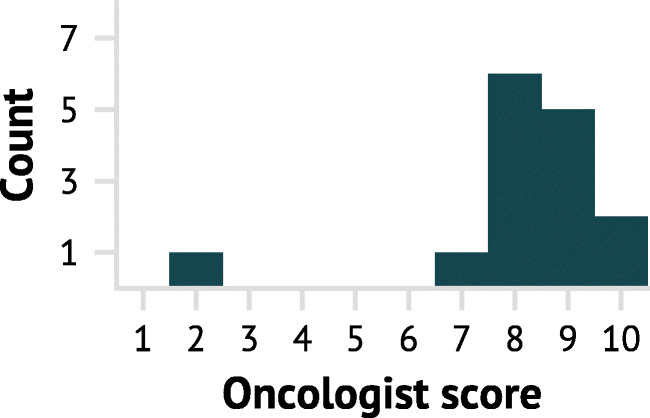
Qualitative evaluation by an experienced oncologist of 15 PET/CT-based CNN delineations, randomly selected from the test cohort. A score of 1 corresponds to a CNN delineation requiring extensive revision whereas a score of 10 corresponds to a CNN delineation that was indistinguishable from a manual delineation

The score distribution of the oncologist’s evaluation of the PET/CT-based CNN delineations for 15 patients in the test cohort is shown in Fig. 4, with performance details given in Table 3. The majority of the CNN delineations were of high quality. Thirteen delineations were scored 8 or higher, indicating that the CNN delineations only required minor modifications by an oncologist. For the two cases receiving a score of 10, the oncologist was unable to decide which delineation was generated by the CNN model and human specialists. Only one case was assessed to a score < 7. This auto-delineation received a score of 2, indicating that major revision was required.

**Table 3:** Performance metrics for 15 randomly selected patients in the test cohort, whose auto-delineated contour (generated by the superior PET/CT model) was evaluated by an experienced oncologist

				HD_95_ (mm)		ASD (mm)		MSD (mm)	
Score	Dice (*%*)	Sens_GT_ (*%*)	PPV_CNN_ (*%*)	SW	PW	SW	PW	SW	PW
*2*	*24*	*0*	*45*	*3.4*	*45*	*1.8*	*13*	*1.8*	*4.9*
7	73	100	17	4.6	30	1.5	4.8	1.1	1.0
8	74	100	17	4.9	33	1.5	4.7	1.0	1.4
8	74	40	29	4.1	17	1.1	2.6	0.57	1.0
*8*	*74*	*100*	*33*	*3.4*	*50*	*1.1*	*5.4*	*0.63*	*1.4*
8	77	100	25	4.6	30	1.4	5.4	0.72	1.4
8	73	67	14	3.9	23	1.1	4.8	0.59	1.4
8	88	100	25	4.9	6.8	1.5	1.8	0.88	0
9	75	100	33	3.4	9.7	1.0	2.1	0.56	1.0
9	85	100	22	4.3	8.1	1.2	1.9	0.50	1.0
9	74	50	56	3.1	3.7	1.1	1.1	0.75	0
9	85	100	38	3.0	5.0	0.73	1.1	0.14	0
9	78	100	33	3.0	42	0.90	5.8	0.40	1.0
10	77	75	75	3.0	4.1	0.86	1.2	0.40	1.0
*10*	*73*	*100*	*33*	*3.6*	*8.8*	*1.2*	*2.5*	*0.74*	*1.0*

**Fig. 5:**
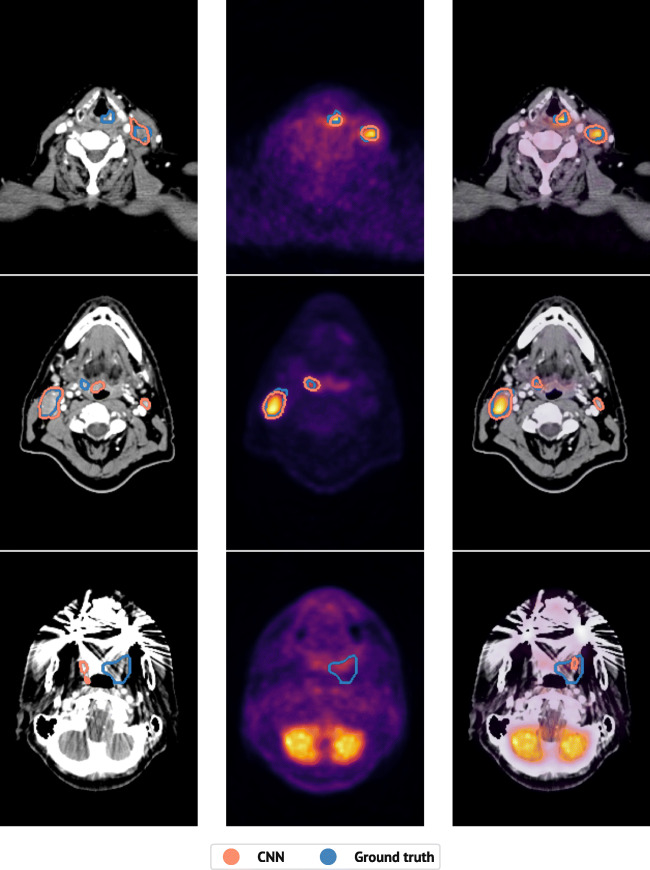
The predicted (*red*) and true (*blue*) delineations for one representative image slice from three different patients in the test cohort. From left to right: CT-based predictions; PET-based predictions; PET/CT-based predictions. From top to bottom, different subjects for whom an experienced oncologist gave the PET/CT-based predictions a qualitative score of 10, 8 and 2, respectively. Performance metrics for the shown patients are marked with italicised text in Table 3. Animations are given in Online Resources 2 (top patient), 3 (middle patient) and 4 (bottom patient)

Dice, Sens_GT_ and PPV_CNN_ are given patient-wise. For split metrics, PW represents the patient-wise metric and SW represents the structure-wise metric. Representative auto-delineations for the italicised rows (patients) are shown in Fig. 5

Figure 5 shows a representative image slice for three patients whose PET/CT delineation was qualitatively assessed by an oncologist. Animations of these delineations are provided in Online Resources 2, 3 and 4 and performance metric details are highlighted in italics in Table 3. The upper row shows a patient for whom the oncologist was unable to differentiate between the PET/CT-based auto-delineation and the ground truth (Online Resource 2). For this patient, both the PET- and the PET/CT-based models performed well, whereas the CT-based model missed the primary tumour in the larynx. The middle row shows a patient for whom the PET/CT-based auto-delineation obtained a qualitative score of 8 (Online Resource 3). The CT-based auto-delineation only identified one structure and contained two false positive structures. PET-based auto-delineation, however, correctly identified both structures. Likewise, the PET/CT-based auto-delineation correctly identified both structures, but included one false positive structure. This false positive structure resulted in a high patient-wise HD_95_ of 50 mm. However, the correctly identified structures had an average structure-wise HD_95_ of 3.4 mm (Table 3), indicating that the CNN model delineated these structures adequately, more in line with the oncologist’s evaluation. The bottom row shows the patient for whom the PET/CT-based auto-delineation obtained a qualitative score of 2 (Online Resource 4). Here, all models, regardless of input-modality, failed at delineating the true structures, likely caused by the strong beam hardening artefacts and low PET-signal.

## Discussion

### Comparison to previous work

To the best of our knowledge, only three previous studies have evaluated the use of CNNs for auto-delineation of the GTV in HNC using multimodal images [8–10]. The PET/MRI-based 3D CNN of Lin et al. [8] and the PET/CT-based 2D CNN described in Huang et al. [9] obtained a median Dice score of 79*%* and a mean Dice score of 74*%*, respectively, for auto-delineation of the primary tumour volume. As in the present study, Guo et al. [10] achieved superior auto-delineation performance of the GTV-T and GTV-N for combined PET/CT input, compared to using single modality CT or PET input. Their PET/CT-based 3D network (Dense Net) resulted in a mean Dice of 71*%*. Similarly, our 2D U-Net obtained a mean Dice of 71*%* for PET/CT-based GTV-T and GTV-N auto-delineation. One notable difference between the present study and the results reported by Guo et al. is the quality of auto-delineations obtained using solely CT images. By only including CT intensities in the range [− 30, 170] HU, we obtained a mean Dice of 56*%*, whereas Guo et al. reported a considerably lower mean Dice of 31*%* using a wider CT window in the range [− 200, 200] HU [10].

Despite differences across imaging modalities in the above studies, the median or mean agreement between CNNs using multimodality input and the expert’s ground truth is above 70*%*. Previous studies conclude that there are considerable interobserver variations in manual HNC target volume delineations [4–7, 17]. In Bird et al. [17], the Dice agreement between five clinicians (three radiation oncologists and two radiologists) was only 56% when delineating the GTV in CT images. Similarly, Gudi et al. [7] found that the Dice agreement between three radiation oncologists was 57% when the GTV was delineated using CT images and 69% when delineated using PET/CT-images. Thus, the interobserver variability of clinicians is similar to the performance of the present CNN-model, which, when evaluated on the test cohort, had an average Dice score of 56% and 71% for the CT and PET/CT-based models, respectively. Furthermore, Lin et al. [8] found that the interobserver and intraobserver variability between oncologists, as well as the contouring time, decreased significantly when CNN-based auto-delineations were used to assist manual delineations, highlighting the possible clinical value of auto-delineation tools.

### Clinical usefulness of CNN-based auto-delineation

Both the quantitative performance metrics and the qualitative oncologist’s evaluation illustrate that despite the moderate amount of training samples and the simple CNN architecture, the models produced delineations of high quality. We observed that the auto-delineations could be useful in RT with just minor to moderate refinements required, such as removing false positive structure, delineating a missing structure, or refining the delineation boundary. We infer this conclusion both from the qualitative scores provided by an expert oncologist as well as the quantitative surface-distance metrics. The average structure-wisesurface distances between true and predicted delineated structures were on the same order of magnitude as the CT resolution ($\sim 1$ mm). Furthermore, structure-wise HD_95_ were on the same order of magnitude as the PET resolution ($\sim 3$ mm). We can, in other words, conclude that the CNN model generated highly accurate auto-delineations, with few exceptions.

The CNN model does, however, exhibit some weaknesses. Some CNN delineated structures are false positives. Moreover, not all ground truth structures are detected. The false positive structures are of minor concern, as most of them are smaller than the resolution of the PET-images. A simple post-processing procedure can easily remove such small structures. In contrast, the lack of sensitivity is more problematic, since all malignant structures should be treated. However, the average Sens_GT_ was 86*%* for the PET/CT model, and all structures were identified for 75*%* of the patients in the test cohort. It was further noted that many of the patients with low CNN performance had beam hardening artefacts on the CT image, leading to slices with little-to-no information from the CT-signal, as can be seen in the bottom row of Fig. 5. This implies that the model worked well for a large portion of the patients, but a small number of patients would still require considerable manual refinements before RT. Lastly, our models were trained and evaluated on images acquired at one single centre. An important next step is an assessment of our models’ generalisability to images stemming from other centres.

### The effect of imaging modality

The CT signal indicates the mass density of tissue. However, we are interested in the properties of soft-tissue tumours and involved lymph nodes, which are only represented in a small section of the CT range. As such, analysing the entire CT range is unnecessary and could even make it harder to find relevant features. This motivated the reduction in dynamic range of the CT images, utilising a soft-tissue window ranging from − 30 to 170 HU.

From a deep learning perspective, such an a priori decrease in dynamic range is not expected to affect model performance to a great extent, as the same transformation can be learned by a two-layer neural network with ReLU activation functions. Nevertheless, our experiments strongly suggest that decreasing the dynamic range of the CT images can have a considerable positive effect on model performance. This increase in performance will likely be less prominent as the dataset size grows, because then, it may be easier for the model to learn the windowing-operation. Further discussion of imaging modalities will, therefore, only consider CT and PET/CT models where the CT images were pre-processed using the given Hounsfield window settings.

When we compare the performance of the models based on their input modality, we notice that the PET signal was essential for discovering the involved lymph nodes correctly. Without the PET-signal, the models, on average, only discovered 60*%* (Sens_GT_) of the manually delineated structures (GTV-T and GTV-N) in the validation cohort. For patients in the test cohort, the CNN model performed worse. The highest performing CT-based CNN-model only managed to identify 53*%* of the manually delineated structures. Conversely, models trained using only PET images and models trained using both PET and CT images, delineated on average 75*%* of the malignant structures for the validation cohort. On the test cohort, the highest performing PET and PET/CT models discovered 77*%* and 86*%*, respectively. Thus, we conclude that the PET signal was crucial for obtaining auto-delineation models with sufficient sensitivity.

A benefit of CT, compared to PET, is its higher spatial resolution. In our experiments, the surface distances between the detected structures and their corresponding ground truth boundaries were smaller for the models that incorporated the CT signal as compared to those without CT input. Hence, the high resolution of the CT was essential to identify the small details and provide an accurate boundary of the structures. Finally, combining the PET signal with the CT signal improved all quantitative performance metrics except for the PPV_CNN_, for most patients. We therefore recommend using a fused PET/CT approach for auto-delineation of head and neck tumours and involved nodes.

### The performance metrics

By including structure-wise performance metrics, as opposed to only voxel- and patient-wise performance metrics, we were able to quantitatively analyse the results in a more in-depth fashion. These structure-wise metrics are meant as a supplement providing additional information on the quality of the auto-delineation, not as a replacement of the well-established and commonly reported metrics, which must be reported to enable cross-study comparisons. Thus, it is the joint information provided by the different types of metrics that we consider useful.

The added information content of the structure-wise metrics is demonstrated in Tables 2 and 3. We see that the patient-wise surface distances are relatively large—especially the HD_95_ metric. However, the corresponding structure-wise distance metrics are much smaller. As these metrics were only calculated for auto-delineated structures that overlapped by more than 50*%* with the ground truth, falsely predicted structures that would otherwise skew the distance-metrics were avoided. Thus, the discrepancy between the patient-wise and the structure-wise distance metrics indicates that the models predict false positive structures. Furthermore, the small structure-wise distance metrics demonstrate that the models performed well for the true structures they actually detected and delineated, thereby indicating how much manual modification is required by the clinician. Likewise, the structure-wise sensitivity, PPV and volumes were very informative as to the types of errors the models make, such as how many true structures they miss, how many false structures they predict and how large these are.

## Conclusions

In summary, we show that CNNs can be used for accurate and precise GTV delineations of HNC using multimodality PET/CT. Furthermore, our proposed structure-wise performance metrics enabled in-depth assessment of CNN predictions and errors, which may facilitate the use of such auto-delineation tools in RT planning.

## Electronic supplementary material

Below is the link to the electronic supplementary material.

(PDF 118 KB)

(MP4 9.71 MB)

(MP4 9.17 MB)

(MP4 4.67 MB)

## Data Availability

Data access requires approval by the Regional Ethics Committee.

## Footnotes

1 These are different β values than those for the *f*_β_ loss.

## Code availability

Code used for model training and evaluation is available at https://github.com/yngvem/EJNMMI-20 and https://github.com/yngvem/mask_stats, respectively.
