# Supplementary material for: Deep learning-based auto-delineation of gross tumour volumes and involved nodes in PET/CT images of head and neck cancer patients
Source: Eur J Nucl Med Mol Imaging. 2021 Feb 9;48(9):2782–92. doi: 10.1007/s00259-020-05125-x (PMC8263429; doi:10.1007/s00259-020-05125-x)
Supplement: Supplementary file 1 — (PDF 118 KB) [file 259_2020_5125_MOESM1_ESM.pdf]

Deep learning based auto-delineation of gross tumour volumes and involved nodes in PET/CT images of head and neck cancer patients

Yngve Mardal Moe · Aurora Rosvoll Groendahl · Oliver Tomic · Einar Dale · Eirik Malinen · Cecilia Marie Futsaether

A Supplementary material

A.1 Dataset statistics

Table A1 shows patient characteristics for the entire patient cohort and the patients included in the training, validation and test cohorts. In addition, Table A2 shows summary statistics for the distribution of structure sizes in the ground truth for the three cohorts. Table A3 shows summary statistics for the number of structures in the ground truth for each patient in the cohorts.

A.2 Architecture

Table A4 shows the architecture of the neural networks. All convolutional layers except the last and all up-convolutional (transposed strided convolution) layers consisted of a (3×3)-(up-)convolution, followed by a ReLU activation function and finally a batch normalisation layer. Up-convolutional layers also included a bilinear interpolation layer after batch normalisation to match the layer before downsampling. The final convolutional layer consisted of a (1×1)-convolution followed by a sigmoidal activation function. All convolutional and up-convolutional layers included a bias-term for each output channel and all convolution-weights were initialised using the normally distributed He-scheme. The code for the experiments are available on GitHub: `https://github.com/yngvem/EJNMMI-2020`.

C.M. Futsaether
Norwegian University of Life Sciences
E-mail: cecilia.futsaether@nmbu.no

Table A1 Patient characteristics.

Characteristic[a]	All patients ($n = 197$)	Train ($n = 142$)	Validation ($n = 15$)	Test ($n = 40$)
**Age [years]**				
Mean	60.3	60.7	58.8	59.4
Range	39.9–79.1	39.9–79.1	43.2–73.7	43.0–77.0
**Sex**				
Female	24.9 %	25.4 %	13.3 %	27.5 %
Male	75.1 %	74.7 %	86.7 %	72.5 %
**TNM[b]**				
T1	9.1 %	9.2 %	6.7 %	10.0 %
T2	39.6 %	39.4 %	40.0 %	40.0 %
T3	23.4 %	23.9 %	20.0 %	22.5 %
T4	27.9 %	27.5 %	33.3 %	27.5 %
N0	23.9 %	25.4 %	6.7 %	25.0 %
N1	11.7 %	12.0 %	13.3 %	10.0 %
N2	60.9 %	58.5 %	80 %	62.5 %
N3	3.6 %	4.2 %	0 %	2.5 %
**AJCC/UICC[b] stage**				
I	1.0 %	1.4 %	0 %	0 %
II	8.6 %	9.2 %	0 %	10.0 %
III	19.8 %	19.7 %	20.0 %	20.0 %
IV	70.1 %	69.0 %	80.0 %	70.0 %
**Tumour site**				
Oral cavity	8.6 %	7.0 %	26.7 %	7.5 %
Oropharynx	72.6 %	73.2 %	60.0 %	75.0 %
Hypopharynx	8.1 %	9.2 %	13.3 %	2.5 %
Larynx	10.7 %	10.1 %	0 %	15.0 %
**GTV-T[c] [cm^3]**				
Mean	25.0	23.9	37.3	24.3
Range	0.8–285.0	0.8–285.0	2.6–247.2	1.4–157.6
**GTV-N[d] [cm^3]**				
Mean	19.3	26.6	37.4	19.5
Range	0.5–276.7	0.5–276.7	2.6–247.2	0.5–76.4

[a] Percentages may not sum to exactly 100 due to rounding.
[b] 7th edition
[c] Gross primary tumour volume
[d] Involved nodal volume (for patients with nodal stage $\geq$ N1)

Table A2 Summary statistics for structure sizes.

		Cohort		
		Train	Validation	Test
Mean	[cm^3]	17	22	16
Standard deviation	[cm^3]	31	43	24
25% percentile	[cm^3]	2.2	2.3	2.3
Median	[cm^3]	7.2	6.3	6.2
75% percentile	[cm^3]	18	27	21
Min	[cm^3]	0.10	0.35	0.31
Max	[cm^3]	28	25	17

Table A3 Summary statistics for the number of structures per patient.

	Cohort		
	Train	Validation	Test
Mean	2.5	2.3	2.3
Standard deviation	1.4	0.86	1.2
25% percentile	2.0	2.0	1.0
Median	2.0	2.0	2.0
75% percentile	3.0	3.0	3.0
Min	1.0	1.0	1.0
Max	10	4.0	6.0

Table A4 Model architecture.

Name	Inputs	Output shape
Conv1	Dataset	$191 \times 265 \times 64$
Conv2	Conv1	$191 \times 265 \times 64$
MaxPool1	Conv2	$95 \times 132 \times 64$
Conv3	MaxPool1	$95 \times 132 \times 128$
Conv4	Conv3	$95 \times 132 \times 128$
MaxPool2	Conv4	$47 \times 66 \times 128$
Conv5	MaxPool2	$47 \times 66 \times 256$
Conv6	Conv5	$47 \times 66 \times 256$
MaxPool3	Conv6	$23 \times 33 \times 256$
Conv7	MaxPool3	$23 \times 33 \times 512$
Conv8	Conv7	$23 \times 33 \times 512$
MaxPool4	Conv8	$11 \times 16 \times 512$
Conv9	MaxPool4	$11 \times 16 \times 1024$
Conv10	Conv9	$11 \times 16 \times 1024$
UpConv1	Conv10	$23 \times 33 \times 512$
Conv11	UpConv1 & Conv8	$23 \times 33 \times 512$
Conv12	Conv11	$23 \times 33 \times 512$
UpConv2	Conv12	$47 \times 66 \times 256$
Conv13	UpConv2 & Conv6	$47 \times 66 \times 256$
Conv14	Conv13	$47 \times 66 \times 256$
UpConv3	Conv14	$95 \times 132 \times 128$
Conv15	UpConv3 & Conv4	$95 \times 132 \times 128$
Conv16	Conv15	$95 \times 132 \times 128$
UpConv4	Conv16	$191 \times 265 \times 64$
Conv17	UpConv4 & Conv2	$191 \times 265 \times 64$
Conv18	Conv17	$191 \times 265 \times 64$
FinalConv	Conv18	$191 \times 265 \times 1$
